# Adult Personality and Its Relationship with Stress Level, Coping Mechanism and Academic Performance among Undergraduate Nursing Students

**DOI:** 10.21315/mjms2022.29.5.12

**Published:** 2022-10-28

**Authors:** Zainah Mohamed, Gurbinder Kaur Jit Singh, Nurulain Shafiqah Dediwadon, Nurul Akma Mohamad Saleh, Nazzatul Nabilah Jupri, Yashnavee Ganesan

**Affiliations:** Department of Nursing, Faculty of Medicine, Universiti Kebangsaan Malaysia, Kuala Lumpur, Malaysia

**Keywords:** students, nursing, personality, adaptation, psychological, academic performance

## Abstract

**Background:**

Stress related to nursing education and clinical placement encounters by students since the beginning of their nursing course has been an issue of concern. This study aims to examine the prevalence of adult personality traits and their relationship with stress levels, coping mechanisms and academic performance among nursing students.

**Methods:**

This cross-sectional study was conducted among 92 nursing students at Universiti Kebangsaan Malaysia (UKM). The Big Five Inventory (BFI), Student Nurse Stress Index and Brief COPE instruments were used to measure the respondents’ personality traits, stress level and coping mechanisms, respectively. Data were analysed using IBM SPSS version 26.

**Results:**

The most prevalent personality trait of the students was openness (mean = 33.58). Conscientiousness (*r* = −0.226, *P* = 0.030) and neuroticism (*r* = 0.326, *P* = 0.002) are significantly related to stress level. Extraversion (*r* = 0.219, *P* = 0.036), conscientiousness (*r* = 0.206, *P* = 0.049) and openness (*r* = 0.219, *P* = 0.036) show significant relationships with the approach coping mechanism, while agreeableness (*r* = −0.257, *P* = 0.013) and neuroticism (*r* = 0.297, *P* = 0.004) show significant relationships with the avoidant coping mechanism. However, no significant relationship was noted between personality traits and academic performance (*r* = 1.000, *P* > 0.05).

**Conclusion:**

Knowledge of ones’ personality traits may benefit students in understanding themselves and in using the best ways to cope with their stress while studying nursing.

## Introduction

Personality traits play an important role in almost every aspect of stress and coping. Personality traits are seen as preparation for thinking or acting similarly in response to various stimuli or situations that are considered stressors (1, 2) and are even able to predict stress level and coping styles (1).

Nursing courses are often perceived as stressful because of the difficulties in education, a longer period of study with a shorter semester break due to the clinical placement and having to deal with patients as early as Year 1. In Malaysia, the nursing course consists of learning theory and clinical posting and nursing students are compulsory to fulfil 85% attendance for lecture hours and 100% attendance for clinical practice placement based on the Standard Criteria for Approval/Accreditation of Nursing Programme 2018 (3). Students in the Bachelor of Nursing programme have an extra workload compared to diploma nursing students due to the additional 30 credits of the core subjects in the nursing curriculum that are compulsory to be achieved (3).

Individuals with neuroticism personality traits are seen as most susceptible to stress compared to extraversion and conscientiousness personality traits, which are less affected by daily stresses. Individuals exhibiting neuroticism tend to use avoidant coping mechanisms, such as venting or denial, rather than problem-focused coping and other active coping strategies, as practiced by other personalities (4). Nursing students are often found to have higher stress levels related to academics and external factors than other healthcare students (5). Studies have shown that the clinical learning environment is perceived as most stressful by students and has the potential to impact the student’s intellectual ability if it remains unresolved (6).

The grasping of nursing knowledge is measured based on students’ performance in both individual and group work, yearly grade point average (GPA) and the nursing board examination (7). Although various factors could influence the students’ academic performance, stress often negatively affects the students’ academic performance, disrupts their learning process and demotivates them to thrive (8). Personality also plays a major part in affecting students’ academic performance, as certain personalities have different learning styles associated with high academic achievers and greater compatibility with their teachers or instructors (9). Many studies have reported the impact of personality traits on students’ academic performance (10, 11). Students with the conscientiousness personality trait were found to score the highest academic performance, then other personalities (10–12), because of the nature of the personality of being hardworking, responsible, dependable and persistent.

Even though many studies have reported the influence of adult personality on nursing students’ academic performance (10–12), most of the studies were conducted in Western countries and the few studies involving Malaysian students were mainly among medical students; little is known about the prevalence of Big Five personality traits among nursing students in Malaysia. Furthermore, the findings from the other studies cannot be generalised to the Malaysian context due to different settings, cultures and backgrounds. Also, previous studies were limited to medical students (4, 13). Therefore, this study aims to identify the prevalence of the Big Five personality traits among Malaysian undergraduate nursing students and the relationship with stress levels, coping mechanisms and academic performance.

Identifying one’s personality traits is beneficial for nursing students in identifying the most effective coping mechanisms for reducing their stress while pursuing a nursing course for better academic performance and motivation to thrive. Moreover, this study can also benefit the nursing fraternity in recognising the different types of personality of the students and provide appropriate attention and psychological help to students based on their personality traits, especially to those with the neuroticism personality who tend to experience difficulty in adapting to the challenging environment of the nursing school. Additionally, the institution can introduce a stress management programme early into the course to help students with neuroticism cope effectively with any stressors.

## Methods

### Sample

This study used a cross-sectional design to measure the prevalence of personality traits and their relationships with the level of stress, coping mechanisms and academic performance among nursing students of Universiti Kebangsaan Malaysia (UKM). The total population was 135 (*n* = 135) nursing students from the Bachelor of Nursing programme from Year 1 through Year 4 during the 2019–2020 session. The required sample size was 100 participants based on Krejcie and Morgan’s (14) formula by excluding those with chronic disease, married and on long medical or study leave. However, only 92 students agreed to participate in this study by completing the questionnaire.

### Instruments

This study was collected using a self-reported questionnaire and extracted data from records. The English language questionnaire consists of four sections: A, B, C and D. Section A consisted of demographic data (matric number, gender, year of study and marital status). Cumulative grade point average (CGPA) was used to measure the student’s academic performance and categorised into two groups: low (0.00–3.09) and high (3.10–4.00).

Section B consisted of the Big Five Inventory (BFI) with 44 items with five subscales: extraversion, agreeableness, conscientiousness, neuroticism and openness with a 5-point Likert scale with option responses of 1 = strongly disagree to 5 = strongly agree (15). Some items were reverse scored. The acceptable reliability of the BFI, with α = 0.72 (16), is consistent with the Cronbach’s alpha of 0.856 in our study.

Section C was adopted from the Student Nursing Stress Index (SNSI), which consists of 22 items with a 5-point Likert scale with option responses of 1 = not stressful to 5 = very stressful (17). The level of stress is indicated by the total score of all subscales. The acceptable reliability of the SNSI with α = 0.70 (18) is supported by good Cronbach α = 0.913 in the present study.

Section D contained the Brief COPE (28 items), which measures students’ coping mechanisms and is comprised of 14 subscales with two items in each subscale. A 4-point Likert scale was used, with 1 = ‘I have not been doing this at all’ to 4 = ‘I have been doing this a lot’ (19). The 14 subscales were categorised into avoidant and approach coping styles, with seven subscales in each. Avoidant coping contains the subscales of denial, substance use, venting, behavioural disengagement, self-distraction, self-blame and humour. Meanwhile, approach coping consists of the subscales of active coping, positive reframing, planning, acceptance, seeking emotional support, seeking informational support and religion. The reliability of Brief COPE was acceptable, with α = 0.80 (20) and further support by α = 0.835 in this study.

### Data Collection Process

Data collection commenced after obtaining ethical approval from the Research Ethics Committee of the UKM Medical Centre. The students from each year were then approached via online meetings. They were given a brief presentation regarding the study. Only participants who completed the consent form beforehand were granted access to the questionnaire. A pilot study was conducted among the nursing students prior to data collection and these students were excluded from the actual study. Online data were collected using Google Forms accompanied by a consent form and an information sheet detailing the purpose of the study, voluntary participation and instructions on completing the questionnaire. To avoid participants becoming tired and bored in answering many questions (94 items) at one time, the questionnaire was divided into two sections, A and B, with an equal number of items in each section.

## Data Analysis

The data was analysed using IBM SPSS version 26.0 and the significance level was set to *P* < 0.05. A descriptive analysis was used to measure the prevalence of the Big Five personality traits by determining the mean and standard deviation. The relationships between personality traits and levels of stress and coping mechanisms were determined using Pearson’s correlation and Spearman’s rank order correlation based on the normality of data distribution. Fisher’s exact test was used to examine the relationship between personality traits and academic performance, as more than 20% of cells had expected counts of less than five.

## Results

In this study, only 92 nursing students participated (response rate of 92%) and most of them were female (*n* = 77, 83.7%), with a small number of male students (*n* = 15, 6.3%). Most of the students were in Year 2 (*n* = 31, 33.7%), followed by Year 1 (*n* = 28, 30.4%), Year 3 (*n* = 19, 20.7%) and Year 4 (*n* = 14, 15.2%). The prevalence of personality traits in descending order was openness, agreeableness, conscientiousness, neuroticism and extraversion (Table 1).

Only conscientiousness and neuroticism personality traits were significantly negatively associated with stress levels (Table 2). Nursing students with personality traits of extraversion, conscientiousness and openness use approach coping mechanisms with significant positive relationships between these variables. However, there was no significant relationship between the personality traits of agreeableness and neuroticism and the approach coping mechanisms (Table 3).

Neuroticism personality trait nursing students significantly used avoidant coping mechanisms, with a *P*-value of 0.004. Both agreeableness and conscientiousness personality traits had significant negative relationships with avoidant coping mechanisms (Table 4).

For analysis purposes, the Big Five personality traits were categorised into two types: adaptive (extraversion, agreeableness, conscientiousness and openness) and maladaptive (neuroticism) (4). Students with adaptive personality traits had the highest rate of low CGPA, at 60.0%. However, two students with maladaptive personality traits shows no difference in their CGPA. Nevertheless, there was no significant relationship between personality traits and academic performance (Table 5).

## Discussion

The findings of this study show that the personality trait of openness was most common among UKM nursing students, whereas extraversion was the least. An openness personality trait person with high intellectual interest and preference for originality and diversity (21) uses creativity, being considerate and impartial in learning and gaining the latest skills and knowledge through new experiences (22). This type of personality trait is suitable for UKM nursing students in learning and gaining new knowledge in their nursing education and adapting to a more diverse environment than the classroom setting during their clinical posting. However, this finding was not supported by a study by Terry et al. (23), whereby agreeableness and neuroticism personality traits were the highest and the lowest personality traits, respectively, among the Bachelor of Nursing students at an Australian university (23). Nevertheless, another study found that year 4 nursing students from the School of Health Professions of an Israeli university scored high for both agreeableness and openness personality traits (24). The differing findings between the studies are probably, as reported, that changes in personality traits are influenced by the environment and age (25). As most of the students in our study were younger (below 23 years old) and at the beginning of their nursing courses (Years 1 and 2), with minimal exposure to nursing as compared to the majority of older age group nursing students in other two studies with a mean age of 35.5 years old (23) and 24.7 years old (24), who were in their final year.

The findings on the levels of stress and personality traits in this study indicate that nursing students with neuroticism personality traits had a higher level of stress. In contrast, in a study in India, both personality traits of neuroticism and extraversion were negatively correlated (*P* < 0.05) with the nursing students’ level of stress (26). Instead, the level of stress was significantly negatively correlated with the conscientiousness personality trait of the nursing students in this study. This finding is supported by a study involving critical care nurses, as the conscientiousness type of personality trait nurses have much lower workplace stress (2). The possible cause could be due to the conscientiousness personality trait being closely related to responsibility and duty (27), and as nursing involves caring for ill patients, nurses who are used to being dutiful and responsible while providing care to patients have learned to overcome their stress.

Regarding the coping mechanisms and personality traits in this study, the findings specify that nursing students tend to adopt both approaches and avoidant coping mechanisms when dealing with their problems. Nursing students with extraversion, openness and conscientiousness personality traits were likely to adopt an approach coping mechanism. This finding was consistent with previous studies (28, 29), in which individuals with these three personality traits showed a significant positive correlation with the approach coping mechanism. This is probably due to the nature of personality traits. As extraversion personality traits individuals often prefer excitement and adventure, they value personal interactions and have a positive outlook, which enables them to handle conflicts and problems optimistically through in-depth assessment (30). These students are generally more adaptive and flexible when facing stressors and use positive reframing to find ways to overcome the stress healthily (28, 31). Additionally, their positive temperaments will often help them to use a coping style that suits both the situation and interpersonal relationships with the people around them (32). Individuals with an openness personality trait and a high level of intellectual curiosity use creative, thoughtful, unbiased and intelligent ways of learning new things and gaining new knowledge from new experiences (22). With conscientious personality traits, individuals are usually well ordered, disciplined, attentive, responsible and acquire a highly in-depth sense of thinking; therefore, both openness and conscientiousness trait individuals tend to engage in problem-focused coping, such as planning and accepting responsibility in dealing with problems (1).

However, the findings in this study indicated that nursing students with neuroticism personality traits preferred avoidant coping mechanisms over approach coping mechanisms. Neuroticism individuals tend to be emotionally unstable, anxious, insecure and depressed (33) and they are likeliest to adopt emotion-focused strategies such as denial, venting and avoiding problems when facing stressors (29). Conversely, nursing students with agreeableness and conscientiousness personality traits evade using avoidant coping mechanisms when dealing with their problems, as a significant negative correlation was noted between these variables in this study.

The finding on the non-significant correlation between personality traits and academic performance in this study was consistent with Nikose et al. (34) but inconsistent with another by Nighute and Sadawarte (35), which reported a significant correlation between the five personality traits and academic performance. Even though personality traits do not strongly influence the students’ academic performance based on the differing findings, acknowledging the various personality traits among the students enables the teachers or instructors to provide support to facilitate the appropriate learning strategies that are closely related to the students’ personality traits for better performance. Although many nursing students with adaptive personality scores had lower CGPAs in this study, direct comparison of whether maladaptive personality is better in affecting the students’ CGPA was not possible with only two students with a maladaptive personality, as shown in the findings.

Future research with a larger sample of nursing students from different institutions allows the generalisation of the findings to other Malaysian nursing students. As environment and age can affect personality traits, an equal proportion of nursing students of younger and older ages from different years of study from various institutions could enable researchers to precisely determine the personality traits of nursing students in Malaysia. The finding on the effect of adaptive personality on academic performance in this study needs further investigation with a larger sample.

## Conclusion

The cross-sectional design used in this study enables us to identify the highest and lowest personality traits of openness and extraction, respectively, among UKM nursing students. Students with neuroticism personality traits scored higher in their stress level and used avoidant coping styles in dealing with their problems. Instead, conscientiousness personality traits were associated with low-stress levels and the approach coping mechanism was notable among other personality traits. Nursing students’ personality traits do not seem to influence their academic performance. However, the findings related to adaptive personality and students’ CGPAs should be accepted cautiously due to the small sample size.

## Figures and Tables

**Table 1:** The prevalence Big Five personality among nursing student (*N* = 92)

Big Five personality traits	Mean	Standard Deviation
Neuroticism	25.43	5.35
Agreeableness	32.75	4.78
Extraversion	24.64	3.87
Conscientiousness	28.51	4.65
Openness	33.58	3.99

**Table 2:** The relationship between Big Five personality traits and level of stress among nursing students of UKM (*N* = 92)

Variable	*r*-value	*P-*value

	Level of stress	
Big Five personality traits		
Extraversion	−0.176^a^	0.094
Agreeableness	−0.139^a^	0.187
Conscientiousness	−0.226^a^	0.030*
Neuroticism	0.326^a^	0.002*
Openness	0.070^b^	0.506

Notes:

a Pearson’s correlation;

b Spearman’s rank correlation;

* sig *P* < 0.05

**Table 3:** Relationship between personality traits and approach coping (*N* = 92)

Variable	*r*-value	*P*-value

	Approach coping	
Big Five personality traits		
Extraversion	0.219^a^	0.036*
Agreeableness	0.193^a^	0.065
Conscientiousness	0.206^a^	0.049*
Neuroticism	−0.097^a^	0.357
Openness	0.219^b^	0.036*

Notes:

a Pearson’s correlation;

b Spearman’s rank correlation;

* sig *P* < 0.05

**Table 4:** Relationship between personality traits and avoidant coping (*N* = 92)

Variable	*r*-value	*P*-value

	Avoidant coping	
Big Five personality traits		
Extraversion	−0.026^a^	0.804
Agreeableness	−0.257^a^	0.013*
Conscientiousness	−0.214^a^	0.041*
Neuroticism	0.297^a^	0.004**
Openness	0.133^b^	0.206

Notes:

a Pearson’s correlation;

b Spearman’s rank correlation;

* sig *P* < 0.05;

** sig *P* < 0.01

**Table 5:** The Big Five personality traits and academic performance (CGPA) (*N* = 92)

Variable		Academic performance (CGPA)		*n* (%)	*P*-value

		Low *n* (%)	High *n* (%)		
Big Five personality	Adaptive	54 (60.0)	36 (40.0)	90 (100.0)	> 0.950
	Maladaptive	1 (50.0)	1 (50.0)	100 (100.0)	
Total		55 (59.8)	37 (40.2)	92 (100.0)	

Notes: Fisher’s exact test; sig *P* < 0.05
